# Is it the picture or is it the frame? An fMRI study on the neurobiology of framing effects

**DOI:** 10.3389/fnhum.2015.00528

**Published:** 2015-10-13

**Authors:** Sarita Silveira, Kai Fehse, Aline Vedder, Katrin Elvers, Kristina Hennig-Fast

**Affiliations:** ^1^Human Science Center, Ludwig-Maximilians-Universität MunichMunich, Germany; ^2^Institute of Medical Psychology, Ludwig-Maximilians-Universität MunichMunich, Germany; ^3^Department of Psychology, University of ViennaVienna, Austria

**Keywords:** visual art, functional magnetic resonance imaging, cognition, aesthetic appreciation, framing

## Abstract

Using functional magnetic resonance imaging (fMRI) we investigated whether a culturally defined context modulates the neurocognitive processing of artworks. We presented subjects with paintings from the Museum of Modern Art (MoMA) in New York, and labeled them as being either from the MoMA or from an adult education center. Irrespective of aesthetic appreciation, we found higher neural activation in the left precuneus, superior and inferior parietal cortex for the MoMA condition compared to the control label condition. When taking the aesthetic preference for a painting into account, the MoMA condition elicited higher involvement of right precuneus, bilateral anterior cingulate cortex (ACC), and temporoparietal junction (TPJ). Our findings indicate that mental frames, in particular labels of social value, modulate both cognitive and affective aspects of sensory processing.

## Introduction

The context, in which sensory input is derived, constitutes a mental frame that determines our relationship to our cultural environment on an implicit level of processing (Pöppel and Bao, 2011). Aiming at unraveling the neurobiological underpinnings of context effects, previous neuroimaging research has demonstrated the impact of knowledge and social or cultural value systems in various domains including decision making, moral reasoning and perception. Evidence highlights the role of prefrontal, temporal, and parietal brain regions in framing effects, which can be associated with working memory, social cognition, and imagery (Bhatt and Camerer, 2005; Gonzalez et al., 2005; Windmann et al., 2006; Deppe et al., 2007; Avram et al., 2014; Fehse et al., 2015). Underlying mechanisms that modulate the evaluation of information have so far been understood as either an influence of emotions on cognition (Mobbs et al., 2006; Brunetti et al., 2014) or cognitive control of emotions, which has found to be represented by inhibitory neural connections between cortical and subcortical brain regions (Camus et al., 2009; Hare et al., 2009). This study aims at a deeper understanding of framing effects on sensory information processing by further elucidating the ways in which social and cultural factors influence or even determine the visual processing of artworks.

Two distinct complementary pathways characterize the processing of visual input. On the one hand visual information is analyzed bottom-up in terms of psychophysical properties; on the other hand prior experiences and expectations take top-down influence on information processing. Accordingly, aesthetic judgment and aesthetic appreciation, which are considered as cognitive and affective outcome of visual arts processing, can be influenced by painting style, color, brush stroke etc. on the one hand, and by expertise, cognitive mastering of the painting or the context in which the painting is perceived on the other hand (Leder et al., 2004). In line with this, it has been shown that the value a perceiving person attaches to an artwork is constrained by anticipations and additional information (Cupchik et al., 1994; Russell, 2003; Leder et al., 2006). In neuroimaging studies, the perception of artworks has been found to involve a complex set of brain areas corresponding to emotional, cognitive and perceptual processes. While in many studies reward-related areas like the ventral striatum, medial prefrontal cortex and anterior insular cortex were associated with aesthetic processes (Kawabata and Zeki, 2004; Cinzia and Vittorio, 2009), in other studies also areas related to cognitive functions like visuo-spatial processes, memory retrieval, or self-related processes were found to be recruited (Fairhall and Ishai, 2008; Cupchik et al., 2009; Silveira et al., 2012; Vessel et al., 2012). The different activation patterns appear to be highly dependent on the behavioral task that was implemented in the study (Vartanian and Skov, 2014). Therefore, aesthetic appreciation and cognitive processing of visual art can be associated with distinct, yet possibly partly overlapping brain networks. Even though brain regions of higher cognitive function like the parietal lobes, the temporoparietal junction (TPJ), or parts of the frontal lobes have been associated with positive aesthetic appreciation (Kawabata and Zeki, 2004; Jacobsen et al., 2006; Cela-Conde et al., 2009; Lutz et al., 2013), it remains a challenge to disentangle neural underpinnings of affective and cognitive aesthetic responses.

Museum venues can generate a framing effect on the aesthetic value of an artwork (Brieber et al., 2014). Previous neuroimaging research provides evidence that a label might modulate the processing of an artwork. Kirk et al. (2009) demonstrated in an functional magnetic resonance imaging (fMRI) study that labeling identical paintings as either art or non-art modulates their processing. Paintings that were labeled as being obtained from a gallery, i.e., from an art-related context, were not only perceived as aesthetically more pleasing, but were also processed with higher activation levels in the medial orbitofrontal cortex (mOFC) and prefrontal cortex, compared to paintings that were labeled as being computer generated. The effect on the neural level was moderated by aesthetic pleasure. Based on their findings, Kirk et al. (2009) suggested that the source of a presented artwork determines expectations and thus, biases an aesthetic judgment. Huang et al. (2011) explored the effects of authenticity for the aesthetic appreciation of art in a neurofunctional study. They demonstrated that assigning paintings as either authentic or fake, regardless of their actual authenticity, had an effect on neural responses. Paintings that were declared as copies were processed with a higher activation in the frontopolar part of the prefrontal cortex and in the middle frontal gyrus. The study highlights that especially naïve observers rely heavily on expert advice in aesthetic matters, indicating the strong social influence on the perception of artworks.

The involvement of non-visual brain areas with respect to context related effects hints at the impact of expectations and knowledge systems on aesthetic appreciation processes. The crucial importance of expectations and artistic knowledge becomes especially evident considering the character of contemporary artworks. Contemporary art cannot be defined by specific visual or optical features. Often contemporary artworks cannot be distinguished from daily life objects. These artworks require specific aesthetic settings and information to be appreciated as artworks and to gain aesthetic value. With the goal to examine the meaning and importance of a specific institutional context for the appreciation of an artwork, this study uses modern paintings as stimulus material. On the basis of previous studies (Kirk et al., 2009; Huang et al., 2011), we further tested in which way information about an exhibition venue serves as a frame that modulates the perception of modern art. While Kirk et al.’s (2009) stimulus set only partly consisted of paintings from the most preeminent art gallery in Denmark, we exclusively used paintings of high social value to reduce the variance in painting quality. We aimed at examining how visual processes are modulated by a label of social reputation as one dimension of the exterior context, thus expanding previous insights on the effects of framing paintings as art and non-art (Kirk et al., 2009) or art and art-copy (Huang et al., 2011). The exhibition venue of an artwork can be seen as a social predictor for its value. It has considerable consequences for the commercial value of the piece and for the artists’ popularity and recognition. This inevitably raises the question whether knowledge about the exhibition venue of an artwork influences aesthetic appreciation and pleasure. We assigned different labels to a selection of paintings from the online database of the Museum of Modern Art (MoMA) in New York, declaring them either as artifacts from the MoMA or from an adult education center. With the MoMA being one of the worlds’ most popular museums (Campbell-Johnston, 2013) we chose a label that reflects the high artistic expertise and aesthetic value of the exhibited artworks. In comparison, an adult education center represents a venue that lacks artistic expertise, recognition and value. Accordingly, we hypothesized that information about the exhibition venue of a painting might frame the perception of art on an implicit level, indicated by neural responses. We were particularly interested in the neural correlates of two different paths of information processing of the presented paintings. Dependent on positive aesthetic appreciation of the paintings we expected increased neural activity for reward-related brain areas when processing paintings with the prestigious MOMA exhibition label. Irrespective of an aesthetic appreciation we rather expected cognition-related brain areas to play a pivotal role in the labeling effect.

## Materials and Methods

### Participants

Seventeen right-handed German-speaking participants (Nine female; mean [μ] 37.0, standard deviation [*SD*] = 6.11 years) with normal or corrected-to-normal vision participated in the study. Informed consent was provided and participants received financial reward. The study was conducted in accordance with the Declaration of Helsinki and approved by the ethics committee of the medical faculty of the LMU Munich.

### Material

In a pilot study, 96 paintings had been evaluated with respect to their exhibition venue. In an online survey, 300 participants (172 female; μ = 34, *SD* = 9.23 years) had to decide whether they believed that the painting is originally located at the MoMA or at an adult education center on a five-point-Likert scale (1 = I’m very sure this painting is originally located at the MoMA; 5 = I’m very sure this painting is originally located at an adult education center). Only those paintings that were not easily assigned to one of the venues (2 < *x* > 4) were selected for the fMRI study. This selection should guarantee that participants could not easily recognize the actual artistic value of the presented painting. All artistic representations were equalized in luminance and resized to a fixed pixel size of 250,000 pixels.

### Procedure

A block design was used with eight blocks per experimental condition and each block comprising three different paintings. Every painting was displayed for 4000 ms with an inter stimulus interval of 250 ms. Each block was followed by the display of a fixation cross for 6000 ms. The order of stimuli and blocks was pseudo-randomized using a stimulus delivery device (Presentation 15.1, Neurobehavioral Systems, Berkeley, CA, USA). While lying in the scanner participants viewed the presented paintings via a mirror attached to the MRI head-coil. To assess explicit aesthetic judgments participants were asked to decide whether they liked the painting or not by pressing one of two buttons of a MRI compatible device (LUMItouch, Photon Control, Burnaby, BC, Canada). Two different labels and thus experimental conditions were chosen to investigate the effect of context specific information for the appreciation of art. The paintings were presented with the label of an exhibition venue, which was either the “MoMA Manhattan” or the “Adult Education Center, Garden City”. For each participant each painting was presented only in one of the two contexts. The paintings were therefore split into two groups and painting-context combinations were randomized between participants; i.e., the same painting that was presented in the context of the exhibition venue “MoMA” to participant A, was presented in the context of the exhibition venue “Adult Education Center” to participant B. Prior to the scanning session participants were provided with information about each of the exhibition venues, which were matched in content (e.g., location, amount of visitors per year, popularity of artists).

The study was conducted with a 3T whole body system at the University Hospital LMU Munich (Achieva, Philips Healthcare, Best, Netherlands). For blood oxygen level dependency (BOLD) imaging T2*-weighted EPI sequence was used (TR = 2500 ms, TE = 30 ms, FA = 80°, 38 axial slices, slice thickness = 3 mm, no inter-slice gap, ascending acquisition, FOV = 448 × 448 mm, matrix = 64 × 64, in-plane resolution = 3 × 3 mm). In total 193 functional volumes were acquired per subject.

### Data Processing and Analysis

Statistical analysis was done with MATLAB (MathWorks, Natick, MA, USA) and with Statistical Package for the Social Sciences (SPSS Statistics 19.0, IBM, Armonk, NY, USA). MRI data were analyzed using Statistical Parametric Mapping software (SPM8, Wellcome Department of Cognitive Neurology, London, UK, http://www.fil.ion.ucl.ac.uk/spm). Anatomical description was done referring to the Automatic Anatomic Labeling (AAL) atlas (Tzourio-Mazoyer et al., 2002) as implemented in the Wake Forest University (WFU) Pickatlas (Advanced NeuroScience Imaging Research Laboratory, Winston-Salem, NC, USA).

The first five functional volumes were discarded to avoid variable effects of blood oxygen saturation on T1 relaxation due to instabilities of the magnetic field. All functional images were 3D motion corrected. In further preprocessing analysis realignment and spatial normalization to the EPI template (Evans et al., 1993) was performed. Spatial smoothing was executed to minimize noise and residual inter-subject differences in anatomy using a Gaussian kernel of 8 mm full width at half maximum (FWHM).

On the first level of statistical analyses a mixed categorical and parametric regression analysis was conducted. Using this approach we modeled two separate polynomial expansions of the functional blood oxygen level dependent signals (Büchel et al., 1998) for each of the two conditions respectively. Every stimulus event, i.e., presentation of a painting, was defined by onset time and 4 s duration. In the 0th order, the two label conditions were modeled by a boxcar function convolved with a hemodynamic response function. In the 1st order expansion, the conditions were additionally scaled in positive and negative aesthetic ratings using a parametric regressor for each condition, and then convolved with a hemodynamic response function. To correct for residual effects of head movements, six motion parameters that were estimated in the preprocessing step were included as covariates for each participant. A high pass filter with a cut-off frequency of 1/128 Hz was used. For all four conditions a mean image was generated for each participant using a contrast weight of 1.

In a second step, two paired samples *t*-tests were calculated accounting for variance between subjects. The statistical models comprised the two conditions (MoMA/adult education center) as within-subjects factors using the individual contrast images generated on the first level either in 0th or 1st order expansion. This procedure is a replication of the methodological approach of Kirk et al. (2009). Significance levels of group contrasts were set *p* < 0.01. Reported significant *p*-values were controlled for alpha error inflation with a cluster-level correction by family wise error *p(FWE)* < 0.05. For the paired *t*-test using mean images of the 1st order polynomial expansion none of the clusters reached that FWE significance. Therefore, we also report uncorrected *p*-values for which we had* a priori* hypotheses.

In analyzing the behavioral data, we used paired *t*-tests for the aesthetic judgments and reaction times. Significance levels were set *p* < 0.05.

## Results

### Behavioral Results

We found no significant differences between the label conditions (MoMA/adult education center) on the behavioral level. In the explicit aesthetic judgments of the paintings, the labeling did not affect aesthetic appreciation of the paintings, *t*_(16)_ = 1.20, *p* = 0.246, with μ = 64, *SD* = 20% positive aesthetic responses for the MoMA and μ = 59, *SD* = 20% positive aesthetic responses for the control label condition. There was also no significant difference in reaction times, *t*_(16)_ = 1.70, *p* = 0.108, with μ = 15, *SD* = 4 s for the MoMA condition and μ = 14, *SD* = 4 s for the control label condition.

### Neuroimaging Results

**Figure 1:**
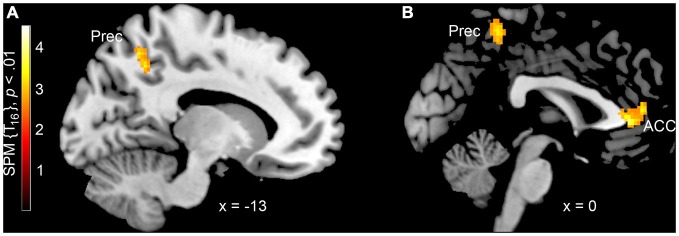
**Higher neural activation when viewing artworks under the label “Museum of Modern Art” compared to a control label condition (A) without and (B) with using aesthetic appreciation as a parametric regressor.** The *x* coordinates are in the MNI space. Prec = precuneus, ACC = anterior cingulate cortex.

Pairwise contrasts were calculated to compare the two different label conditions to each other. In the 0th order expansion, there was higher activation for the MoMA condition compared to the control label condition in the left precuneus, superior and inferior parietal cortex (Table 1A; Figure 1A). The reverse contrast did not reveal regions of significantly higher activation for the control label condition.

**Table 1:** **Neural correlates of differential processing of visual art under label conditions**.

	Coordinates			*z*-statistics	Size in voxels
Brain region	*x*	*y*	*z*
**(A)** Main effect MoMA > control label
Cluster					959
L precuenus	−18	−54	48	3.68	
L superior parietal cortex	−40	−50	54	3.19	
L inferior parietal cortex	−40	−60	52	3.37	
**(B)** MoMA > control label modulated by aesthetic ratings (positive > negative aesthetic judgments)
Anterior cingulate cortex	−4	38	2	3.61	256
Precuneus	4	−44	60	3.17	124
L middle temporal gyrus	−50	−62	18	3.28	332
R superior temporal gyrus	−54	−40	14	3.25	375

*Note: Right (R) or left (L) hemisphere. The x, y and z coordinates are in the MNI space, z-values of t-test. 2 × 2 × 2 mm voxel size*.

When aesthetic rating was included as a parametric regressor, i.e., when taking aesthetic appreciation of the presented paintings into account, there was a higher activation for the MoMA label. For positive aesthetic ratings there was an increase of metabolism in the bilateral anterior cingulate cortex (ACC), the right precuneus and in the bilateral TPJ (Table 1B; Figure 1B). In both models, the reverse contrast did not reveal higher activation for the control condition label.

## Discussion

We addressed the question whether the exhibition venue and its reputation can set a mental frame that modulates the neural correlates of aesthetic perception and whether this effect is dependent on the aesthetic appreciation of paintings. On the level of neurofunctional processes, the assignment of different labels modulated the processing of identical paintings on a neural level. However, there were no significant context related effects on average aesthetic ratings. It is well known that explicit and implicit measures are sensitive to different levels of mental operations. In art perception, the former has often been referred to as the intellectual appreciation of an artwork (Cupchik, 1999) and might also be undermined by a social desirability bias (Edwards, 1957; Temme and Gieszen, 1995). However, when taken previous studies into account it is surprising that no effect on the explicit level could be found. In this regard, we couldn’t replicate the finding that different exhibition contexts modulate the appreciation of art. This might be traced back to differences in the response measure of aesthetic appreciation. While Kirk et al. (2009) used a Likert scale, we used a dichotomous preference ratings. The nature of forced choice paradigms provokes an artificial decision to express either positive or negative attitude towards a stimulus. However, we chose this behavioral measurement as aesthetic appreciation has been characterized by categorically differential response patterns between favored and depreciated paintings rather than a continuous increase or decrease of responses from negative to positive on the neural level (Vessel et al., 2012). In general, aesthetic appreciation cannot only be represented on either a continuum or a categorical difference between liking to not-liking. Especially when is comes to aesthetics, subjective aspects play a pivotal role in appreciation and preference choices. Even though in the current study, we only investigated one aspect of the exterior context as a modulating factor of brain responses, we want to point out that the aesthetics cannot be externalized, but are also a property of the viewer’s response to the artwork. On the basis of our results, we thus can’t make conclusions on the subjective quality of visual arts perception and aesthetic appreciation, but only framing effects that are related to contextual modulations of visual perception.

An effect of the given contexts was investigated by comparing the processing of paintings under two different label conditions. We found stronger BOLD signals for the MoMA label compared to the control label condition. However, brain regions corresponding to this label effect differed dependent on liking of the respective painting, thus supporting findings of Kirk et al. (2009) However, the brain regions involved in this framing effect suggest different underlying processes; while Kirk et al. (2009) demonstrate that common reward-related brain areas are recruited with respect to positive aesthetic appreciation, we particularly found the precuneus to be engaged in contextual changes both with and without taking positive aesthetic judgments into account. This cardinal difference in findings between the two studies might be explained by the difference in response measure as Kirk et al. (2009) measured brain activity reflecting a graded liking response. Even though also the recruitment of parietal regions has previously been linked to positive aesthetic judgments in general (Kawabata and Zeki, 2004; Vartanian and Goel, 2004; Cela-Conde et al., 2009; Cupchik et al., 2009; Huang et al., 2011; Lacey et al., 2011), activation of the precuneus might indicate aspects of a framing effect that are less likely related to aesthetic appreciation alone. This assumption also corresponds to the lack of an effect on the explicit level of aesthetic appreciation.

In this regard, the spatial dissociation of parietal activation patterns between the two statistical models might be representative of different underlying processes. While involvement of precuneus independent of aesthetic ratings is located in the left hemisphere, for aesthetically determined responses it is located in the right hemisphere. The latter one can be seen as a confirmation of previous findings, where the right hemisphere of the parietal lobe has continuously been associated with positive aesthetic ratings (Lutz et al., 2013). It also corresponds to findings of Huang et al. (2011) that the right precuneus is involved in a framing effect of processing visual art as either authentic or fake. The precuneus has been functionally ascribed to a set of diverse cognitive processes that can be broadly divided into episodic memory retrieval, visuo-spatial imagery, attentional, and self-related operations (Cavanna and Trimble, 2006). In the context of aesthetic visual information processing, particularly the notion of precuneus being associated with personal relevance has been supported by previous research (Silveira et al., 2012; Vessel et al., 2012). Trying to disentangle and identify functional ascriptions of precuneus activation, structural subsections have been suggested that divide the precuenus anatomically in an anterior, central, and posterior part (Margulies et al., 2009). In the current study, modulation of BOLD signals were found in the cognitive/associative central precuneus. This subsection of the precuneus has been shown to elicit resting-state functional connectivity with parietal regions and the superior temporal sulcus as well as with the medial frontal cortex (brodman area 32; Petrides and Pandya, 1984; Margulies et al., 2009). In particular, for framing effects modulated by aesthetic appreciation, we found a simultaneous activation pattern in the ACC, TPJ, and central precuneus, corroborating with the notion of cognitive functions being involved in framing effects.

In a broader sense, our results provide insight into the neurobiology of framing effects in general. Previous research has provided evidence that temporal and parietal brain regions are involved in framing effects, i.e., modulated by social norms and values (Bhatt and Camerer, 2005; Gonzalez et al., 2005). In particular, temporal structures like the TPJ might indicate processes related to social cognition (Gallagher and Frith, 2003; Saxe and Kanwisher, 2003). Our study highlights the predominant role of precuneus in top-down processing. Offering a promising taxonomy of framing effects, Levin et al. (1998) coined the term “attribute framing” for phenomena that can be related to an attribute of a stimulus and its positive or negative valence. In particular, effects of brands can be seen as exemplary for attribute framing. Research on the neural correlates of brands is still limited and somewhat ambiguous (Ariely and Berns, 2010; Plassmann et al., 2012). Evidence is provided that involvement of precuneus might be relevant in effects of luxury brands (Schaefer and Rotte, 2007). In terms of exhibition venues, the MoMA might be seen as a fairly strong brand. Thus, our results support previous findings that attribute framing effects of labels with a positive connotation in terms of social popularity as compared to those labels with lack of social reputation are related to the precuneus. This also corroborates with the notion that effects of familiar brands are associated with personal relevance and thus, self-relatedness (Santos et al., 2012; Bruce et al., 2014).

## Conclusion

Taken together, our study provides evidence that expectations and knowledge systems modulate the perception of visual art. This indicates the importance of psychological and social contexts for the appreciation and aesthetic experience of art. Even though all paintings used in the study can be considered as “good art”, the assignment of a label positively influenced the aesthetic value on an implicit level. The apparent modulations of neural activation reflect changes in top-down processes of visual perception. Ultimately, attaching aesthetic value to an artwork seems to be determined by social norms. It is not only the picture we admire, it is also the frame.

## Author contributions

KF designed the study. KE and SS performed the fMRI-measurement. SS analyzed the data. KF, SS and AV wrote the manuscript. KH-F revised the manuscript. SS edited the study.

## Conflict of Interest Statement

The authors declare that the research was conducted in the absence of any commercial or financial relationships that could be construed as a potential conflict of interest.

## References

[B2] Ariely D. Berns G. (2010). Neuromarketing: the hope and hype of neuroimaging in business. Nat. Rev. Neurosci. 11, 284–292. 10.1038/nrn2795 20197790 PMC2875927

[B55] Avram M. Hennig-Fast K. Bao Y. Pöppel E. Reiser M. Blautzik J.. (2014). Neural correlates of moral judgments in first- and third-person perspectives: implications for neuroethics and beyond. BMC Neurosci. 15:39. 10.1186/1471-2202-15-39 24742205 PMC3991864

[B4] Bhatt M. Camerer C. F. (2005). Self-referential thinking and equilibrium as states of mind in games: fMRI evidence. Games Econ. Behav. 52, 424–459. 10.1016/j.geb.2005.03.007

[B56] Brieber D. Nadal M. Leder H. Rosenberg R. (2014). Art in time and space: context modulates the relation between art experience and viewing time. PLoS One 9:e99019. 10.1371/journal.pone.0099019 24892829 PMC4043844

[B5] Bruce A. S. Bruce J. M. Black W. R. Lepping R. J. Henry J. M. Cherry J. B. C.. (2014). Branding and a child’s brain: an fMRI study of neural responses to logos. Soc. Cogn. Affect. Neurosci. 9, 118–122. 10.1093/scan/nss109 22997054 PMC3871732

[B6] Brunetti M. Perrucci M. G. Di Naccio M. R. Ferretti A. Del Gratta C. Casadio C.. (2014). Framing deductive reasoning with emotional content: an fMRI study. Brain Cogn. 87, 153–160. 10.1016/j.bandc.2014.03.017 24747514

[B57] Büchel C. Holmes A. P. Rees G. Friston K. J. (1998). Characterizing stimulus-response functions using nonlinear regressors in parametric fMRI experiments. Neuroimage 8, 140–148. 10.1006/nimg.1998.0351 9740757

[B7] Campbell-Johnston R. (2013). The world’s 50 greatest galleries. The Times. Available online at: http://www.thetimes.co.uk

[B8] Camus M. Halelamien N. Plassmann H. Shimojo S. O’Doherty J. Camerer C.. (2009). Repetitive transcranial magnetic stimulation over the right dorsolateral prefrontal cortex decreases valuations during food choices. Eur. J. Neurosci. 30, 1980–1988. 10.1111/j.1460-9568.2009.06991.x 19912330

[B9] Cavanna A. E. Trimble M. R. (2006). The precuneus: a review of its functional anatomy and behavioural correlates. Brain 129, 564–583. 10.1093/brain/awl004 16399806

[B10] Cela-Conde C. J. Ayala F. J. Munar E. Maestú F. Nadal M. Capò M. A.. (2009). Sex-related similarities and differences in the neural correlates of beauty. Proc. Natl. Acad. Sci. U S A 106, 3847–3852. 10.1073/pnas.0900304106 19237562 PMC2656168

[B16] Cinzia D. D. Vittorio G. (2009). Neuroaesthetics: a review. Curr. Opin. Neurobiol. 19, 682–687. 10.1016/j.conb.2009.09.001 19828312

[B11] Cupchik G. C. (1999). The thinking-I and the being-I in psychology of the arts. Creat. Res. J. 12, 165–173. 10.1207/s15326934crj1203_1

[B12] Cupchik G. C. Shereck L. Spiegel S. (1994). The effects of textual information on artistic communication. Vis. Arts Res. 20, 62–78.

[B13] Cupchik G. C. Vartanian O. Crawley A. Mikulis D. J. (2009). Viewing artworks: contributions of cognitive control and perceptual facilitation to aesthetic experience. Brain Cogn. 70, 84–91. 10.1016/j.bandc.2009.01.003 19223099

[B15] Deppe M. Schwindt W. Pieper A. Kugel H. Plassmann H. Kenning P.. (2007). Anterior cingulate reflects susceptibility to framing during attractiveness evaluation. Neuroreport 18, 1119–1123. 10.1097/wnr.0b013e3282202c61 17589310

[B18] Edwards A. L. (1957). The Social Desirability Variable in Personality Assessment and Research. New York, NY: The Dryden Press.

[B19] Evans A. C. Collins D. L. Mills S. R. Brown E. D. Kelly R. L. Peters T. M. (1993). 3D statistical neuroanatomical models from 305 MRI volumes. Proc. IEEE Nucl. Sci. Symp. Med. Imaging 3, 1813–1817. 10.1109/nssmic.1993.373602

[B20] Fairhall S. Ishai A. (2008). Neural correlates of object indeterminacy in art compositions. Conscious. Cogn. 17, 923–932. 10.1016/j.concog.2007.07.005 17714955

[B58] Fehse K. Silveira S. Elvers K. Blautzik J. (2015). Compassion, guilt and innocence: an fMRI study of responses to victims who are responsible for their fate. Soc. Neurosci. 10, 243–252. 10.1080/17470919.2014.980587 25398075

[B23] Gallagher H. L. Frith C. D. (2003). Functional imaging of ’theory of mind’. Trends Cogn. Sci. 7, 77–83. 10.1016/s1364-6613(02)00025-6 12584026

[B24] Gonzalez C. Dana J. Koshino H. Just M. (2005). The framing effect and risky decisions: examining cognitive functions with fMRI. J. Econ. Psychol. 26, 1–20. 10.1016/j.joep.2004.08.004

[B25] Hare T. Camerer C. Rangel A. (2009). Self-control in decision-making involves modulation of the vmPFC valuation system. Science 324, 646–648. 10.1126/science.1168450 19407204

[B26] Huang M. Bridge H. Kemp M. Parker A. (2011). Human cortical activity evoked by the assignment of authenticity when viewing works of art. Front. Hum. Neurosci. 5:134. 10.3389/fnhum.2011.00134 22164139 PMC3225016

[B27] Jacobsen T. Schubotz R. I. Höfel L. Cramon D. Y. (2006). Brain correlates of aesthetic judgment of beauty. Neuroimage 29, 276–285. 10.1016/j.neuroimage.2005.07.010 16087351

[B29] Kawabata H. Zeki S. (2004). Neural correlates of beauty. J. Neurophysiol. 91, 1699–1705. 10.1152/jn.00696.2003 15010496

[B30] Kirk U. Skov M. Hulme O. Christensen M. Zeki S. (2009). Modulation of aesthetic value by semantic context: an fMRI study. Neuroimage 44, 1125–1132. 10.1016/j.neuroimage.2008.10.009 19010423

[B31] Lacey S. Hagtvedt H. Patrick V. M. Anderson A. Stilla R. Deshpande G.. (2011). Art for reward’s sake: visual art recruits the ventral striatum. Neuroimage 55, 420–433. 10.1016/j.neuroimage.2010.11.027 21111833 PMC3031763

[B32] Leder H. Belke B. Oeberst A. Augustin D. (2004). A model of aesthetic appreciation and aesthetic judgments. Br. J. Psychol. 95, 489–508. 10.1348/0007126042369811 15527534

[B33] Leder H. Carbon C. C. Ripsas A. L. (2006). Entitling art: influence of title information on understanding and appreciation of paintings. Acta Psychol. (Amst) 121, 176–198. 10.1016/j.actpsy.2005.08.005 16289075

[B34] Levin I. Schneider S. Gaeth G. (1998). All frames are not dreated equal: a typology and critical analysis of framing effects. Organ. Behav. Hum. Decis. Process. 76, 149–188. 10.1006/obhd.1998.2804 9831520

[B35] Lutz A. Nassehi A. Bao Y. Pöppel E. Sztrókay A. Reiser M.. (2013). Neurocognitive processing of body representations in artistic and photographic images. Neuroimage 66, 288–292. 10.1016/j.neuroimage.2012.10.067 23123681

[B36] Margulies D. S. Vincent J. L. Kelly C. Lohmann G. Uddin L. Q. Biswal B. B.. (2009). Precuneus shares intrinsic functional architecture in humans and monkeys. Proc. Natl. Acad. Sci. U S A 106, 20069–20074. 10.1073/pnas.0905314106 19903877 PMC2775700

[B37] Mobbs D. Weiskopf N. Lau H. C. Featherstone E. Dolan R. J. Frith C. D. (2006). The kuleshov effect: the influence of contextual framing on emotional attributions. Soc. Cogn. Affect. Neurosci. 1, 95–106. 10.1093/scan/nsl014 17339967 PMC1810228

[B39] Petrides M. Pandya D. N. (1984). Projections to the frontal cortex from the posterior parietal region in the rhesus monkey. J. Comp. Neurol. 228, 105–116. 10.1002/cne.902280110 6480903

[B40] Plassmann H. Ramsøy T. Milosavljevic M. (2012). Branding the brain: a critical review and outlook. J. Consum. Psychol. 22, 18–36. 10.1016/j.jcps.2011.11.010

[B41] Pöppel E. Bao Y. (2011). “Three modes of knowledge as basis for intellectual cognition and communication: a theoretical perspective,” in Culture and Neural Frames of Cognition and Communication, eds Han S. Pöppel E. (Heidelberg: Springer), 215–231.

[B42] Russell P. A. (2003). Effort after meaning and the hedonic value of paintings. Br. J. Psychol. 94, 99–110. 10.1348/000712603762842138 12648392

[B43] Santos J. P. Moutinho L. Seixas D. Brandão S. (2012). Neural correlates of the emotional and symbolic content of brands: a neuroimaging study. J. Customer Behav. 11, 69–93. 10.1362/147539212x13286273975319

[B44] Saxe R. Kanwisher N. (2003). People thinking about thinking people: the role of the temporo-parietal junction in “theory of mind”. Neuroimage 19, 1835–1842. 10.1016/s1053-8119(03)00230-1 12948738

[B45] Schaefer M. Rotte M. (2007). Thinking on luxory or pragmatic brand products: brain responses to different categories of culturally based brands. Brain Res. 1165, 98–104. 10.1016/j.brainres.2007.06.038 17655834

[B46] Silveira S. Graupmann V. Frey D. Blautzik J. Meindl T. Reiser M.. (2012). Matching reality in the arts: self-referential neural processing of naturalistic compared to surrealistic images. Perception 41, 569–576. 10.1068/p7191 23025160

[B47] Temme J. E. Gieszen C. (1995). Contrast effects and social desirability in art appreciation. Empir. Stud. Arts 13, 171–181. 10.2190/7eha-jdc4-uvbj-h1rv

[B48] Tzourio-Mazoyer N. Landeau B. Papathanassiou D. Crivello F. Etard O. Delcroix N.. (2002). Automated anatomical labeling of activations in SPM using a macroscopic anatomical parcellation of the MNI MRI single-subject brain. Neuroimage 15, 273–289. 10.1006/nimg.2001.0978 11771995

[B49] Vartanian O. Goel V. (2004). Neuroanatomical correlates of aesthetic preference of paintings. Neuroreport 15, 893–897. 10.1097/00001756-200404090-00032 15073538

[B50] Vartanian O. Skov M. (2014). Neural correlates of viewing paintings: evidence from a quantitative meta-analysis of functional magnetic resonance imaging data. Brain Cogn. 87, 52–56. 10.1016/j.bandc.2014.03.004 24704947

[B51] Vessel E. Starr G. Rubin N. (2012). The brain on art: intense aesthetic experience activates the default mode network. Front. Hum. Neurosci. 6:66. 10.3389/fnhum.2012.00066 22529785 PMC3330757

[B52] Windmann S. Kirsch P. Mier D. Stark R. Walter B. Güntürkün O.. (2006). On framing effects in decision making: linking lateral versus medial orbitofrontal cortex activation to choice outcome processing. J. Cogn. Neurosci. 18, 1198–1211. 10.1162/jocn.2006.18.7.1198 16839292

